# Cancer classification based on chromatin accessibility profiles with deep adversarial learning model

**DOI:** 10.1371/journal.pcbi.1008405

**Published:** 2020-11-09

**Authors:** Hai Yang, Qiang Wei, Dongdong Li, Zhe Wang

**Affiliations:** 1 Department of Computer Science and Engineering, East China University of Science and Technology, Shanghai, PR China; 2 Department of Molecular Physiology & Biophysics, Vanderbilt University, Nashville, Tennessee, United States of America; 3 Vanderbilt Genetics Institute, Vanderbilt University, Nashville, Tennessee, United States of America; Queen's University, CANADA

## Abstract

Given the complexity and diversity of the cancer genomics profiles, it is challenging to identify distinct clusters from different cancer types. Numerous analyses have been conducted for this propose. Still, the methods they used always do not directly support the high-dimensional omics data across the whole genome (Such as ATAC-seq profiles). In this study, based on the deep adversarial learning, we present an end-to-end approach ClusterATAC to leverage high-dimensional features and explore the classification results. On the ATAC-seq dataset and RNA-seq dataset, ClusterATAC has achieved excellent performance. Since ATAC-seq data plays a crucial role in the study of the effects of non-coding regions on the molecular classification of cancers, we explore the clustering solution obtained by ClusterATAC on the pan-cancer ATAC dataset. In this solution, more than 70% of the clustering are single-tumor-type-dominant, and the vast majority of the remaining clusters are associated with similar tumor types. We explore the representative non-coding loci and their linked genes of each cluster and verify some results by the literature search. These results suggest that a large number of non-coding loci affect the development and progression of cancer through its linked genes, which can potentially advance cancer diagnosis and therapy.

## Author summary

A few methods have been developed to leverage omics data (e.g., iCluster) to solve cancer classification. However, these omics data always focus on the coding regions (2% of the human genome). Cancer classification methods based on the high-dimensional raw data across the whole genome are rare. Our approach addressed crucial and fundamental limitations in existing approaches. ClusterATAC used adversarial learning to handle the limited but high-dimensional omics data. Feature selection techniques are used to analyze the essential non-coding loci and their regulatory genes for each cluster. The outcome can lead to a deeper understanding of the regulatory mechanisms that lead to cancer development and progression. We successfully obtained 22 cancer subgroups form the ATAC-seq profiles of 401 TCGA samples. We observed that most subgroups follow the 'Cell-of-Origin' pattern, consistent with the recent study. There were significant differences between the 22 clusters. More than 70% of the subgroups were homogeneous for a single cancer type. We identified the representative loci and the corresponding regulatory genes of each cluster. We found that these loci and genes are always tumor-specific and responsible for the occurrence and development of the related tumor.

## Introduction

Cancer is a heterogeneous complex disease that poses a severe threat to human health and can occur in most organs of the human body [1]. The character of cancer is the infinite proliferation of cells, invasion of normal tissues, and transfer to distant organs [2]. It is essential to determine the cancer types of patients in cancer treatment and select clinical and drug treatment options based on the classification results [3]. The traditional cancer pathology classification method is based on the tissue-histology information and has achieved great success. However, this classification method ignores the commonality of molecular profiles in different types of cancer patients, causing challenges to interpret the molecular mechanisms on some individual tumors and limiting the development of new treatment modalities [4]. Recently, to more accurately diagnose cancer and formulate treatment plans, with the rapid accumulation of cancer omics profiles, molecular classification based on individual molecular platforms across tissues has become critical [5]. Accurate classification results can lead to the discovery of pathogenic mechanisms, cancer driver genes, or deleterious mutations. They can help with the development of precision medical therapy, which has become a hot issue in cancer treatment.

With the rapid development of next-generation genome sequencing technology, large cancer research projects, such as The Cancer Genome Atlas (TCGA) [6] and the International Cancer Genome Consortium (ICGC) [7], have published numerous different types of genomic data, which exceedingly promote the development of cancer genomics research. With these molecular profiles, several analyses achieve meaningful results. In 2014, based on the integration of TCGA multi-omics data, the multiplatform study across 12 cancer types (Pan-Cancer-12) suggested that the molecular classification results are significantly different from the pathological classification results [5]. In 2018, TCGA published multiple genomic data from more than 10,000 patients across 33 different types of cancer [8–14]. These data include 3.6 million somatic mutation data from various cancer centers and other massive omics data (such as gene expression, methylation, protein expression, copy number). An integrative classification analysis reported 28 different clusters (28-iClusters solution). The study found that the ‘Cell-of-Origin’ pattern determines the main results. Other factors influence the clustering results (such as the copy-number aberrations and immune features), which led to partially mixed tumor groups in the clustering (including pan-kidney, pan-squamous, and immune-related mixture category). At the same time, another study collected the assay for transposase accessible chromatin with sequencing (ATAC-seq) data from 410 TCGA samples and conducted the clustering analysis [15]. This study focused on the effects of chromatin accessibility in the genome-wide regions on various types of tumors and found 18 distinct molecular clusters (highly consistent with the 28-iClusters solution). These analyses explore the molecular classification across multiple tumor types and suggest future directions for cancer therapy.

The ATAC-seq technology is introduced into cancer profiles analysis since it can mark the open chromatin sites and predict transcription factor (TF) binding sites across the whole genome [16]. However, the bottleneck of using ATAC-seq data in clustering is that the data sample size is small, and the dimension of data is very high. The current clustering methods (such as iCluster [17], NEMO [18], MultiNMF [19]) are mainly developed for integrating different types of genomic data but not focus on processing the high-dimensional complex data. The DensityPeakCluster method used in the previous study [15] can handle ATAC-seq profiles. However, it requires manual setting of model parameters based on the distribution of input to determine the number of clusters, which causes inconvenience for large-scale analyses. The development of clustering algorithms for high-dimensional omics data remains a challenge [20]. They are required to address the following requirements: automatically handle high-dimensional input data and determine the number of clusters; achieve stable clustering performance; explain the results thoroughly; revealed the influence of non-coding regulatory factors on the clustering results.

Recent advances in the artificial intelligence (AI) field allows deep learning applied to a large number of research fields. With the development of novel modeling techniques, more and more types of neural networks have been proposed, such as multilayer perceptron, convolutional neural network, auto-encoder, recurrent neural network, and generative adversarial network. Deep learning has not only gained enormous success in image recognition, object detection, speech recognition, natural language understanding but also gradually began to be applied creatively in molecular biology-related studies (such as population genetic inference [21], microRNA targets prediction [22], drug discovery [23]). Recently, deep learning technology has been utilized to computer-aided diagnosis (CAD) and has made a tremendous breakthrough in the major cancer types (such as breast cancer, lung cancer, skin cancer, pancreatic cancer, brain cancer, colon cancer) [24]. However, the applications of deep learning for the cancer molecular profiles analyses are relatively rare. It is mainly because most deep learning networks are based on supervised learning and require a large number of accurately labeled samples for the model training. Due to the heterogeneity and complexity of cancer, many molecular mechanisms are mysterious. Entirely accurate annotation of the molecular data is very scarce. Moreover, the collection of molecular data is costly. Although high-throughput sequencing technologies are rapidly developing, it is complicated to collect large-scale samples for sufficient neural network training. The results obtained are often tough to understand and explain due to the nonlinearity and complexity of neural networks. These bottlenecks limit the use of deep learning for the analysis of molecular cancer data. In particular, the use of the neural networks in the cancer classification task is challenging since it is an unsupervised learning task with no known labels.

**Fig 1:**
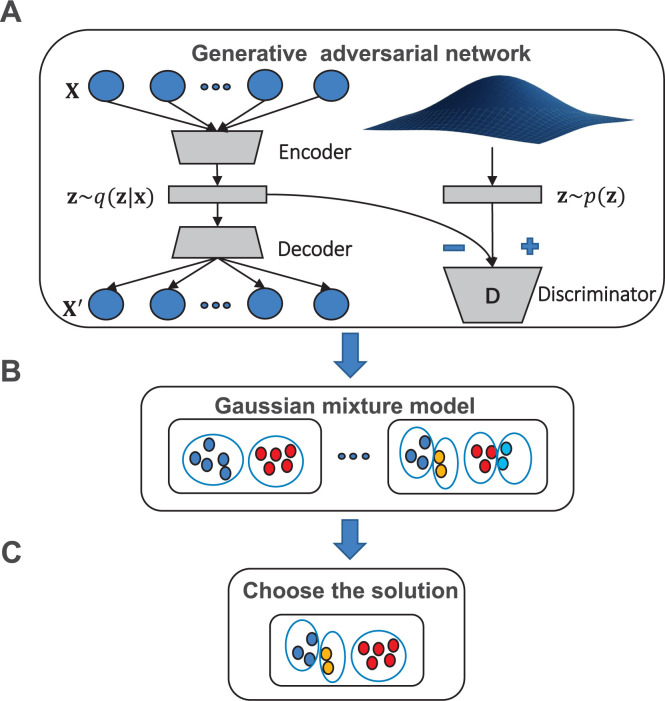
Overview of the ClusterATAC framework. (A) Features extracted from the original ATAC-seq data. (B) GMM identified subgroup-solutions with different cluster number K. (C)Use the Davies-Bouldin index to choose the most suitable solution.

To solve the challenges of deep learning in the cancer classification, and explore the relationship between the non-coding regulatory elements and distinct clusters, we develop the ClusterATAC framework based on the ATAC-seq data. ClusterATAC consists of two modules, Encoder-GAN and Gaussian Mixture Model (GMM) (Fig 1). Encoder-GAN uses the Generative Adversarial Network (GAN) [25] architecture for the model training process, and GMM is applied to the outputs of Encoder-GAN for the clustering process. To prove that ClusterATAC can handle high-dimensional omics data, we collected ATAC-seq data of 401 pan-cancer samples and RNA-seq data of 1031 breast cancer samples and constructed two benchmark data sets. On these two datasets, ClusterATAC obtained stable and interpretable clustering results. Next, to reveal our approach has excellent performance, we compared the performance of ClusterATAC with four state-of-art methods on the two data sets. Then, to explore the association between non-coding regions and the clustering schemes derived by ClusterATAC, we focused on the ATAC-seq data set and performed the heatmap analysis of ClusterATAC and other approaches to fully understand the clustering schemes of ClusterATAC. We further use feature selection techniques to analyze the essential non-coding loci for each subgroup and the regulatory genes they linked and conducted literature searches to support our findings. Based on the above, we believe that the 22-cluster solution generated by ClusterATAC can expand our understanding of the role of non-coding loci in tumor development.

## Results

### Interpretation of the clustering results of ClusterATAC

To illustrate that ClusterATAC can achieve stable and reasonable results in the clustering of ultra-high dimension data, we constructed two tumor datasets. The first dataset comprised 401 TCGA samples with complete ATAC-seq data and clinical profiles (data dimension is about 500,000). The ATAC-seq profiles were obtained from the previous analysis [15]. The second dataset included RNA-seq profiles of 1031 BRCA samples in the TCGA database [6] (data dimension is about 20,000).

We applied ClusterATAC on the ATAC-seq data to discover the distinct patterns of samples across 23 cancer types. During the clustering process, we used the normalized ATAC-seq peak scores as the feature to represent the samples. Since each sample's feature dimension is very high (562,709 peaks), to avoid overfitting, we developed the Encoder-GAN to handle the ATAC-seq and obtained the latent variables corresponding to the individual. We introduced GAN since it can enhance the representation ability of the deep learning approach. After the GAN training, the model successfully extracted the features from the original data as the input of the clustering process (S1 Text). Based on the nonlinearly encoded features, we performed molecular subtyping with GMM clustering. The Davies-Bouldin index [26] was applied to facilitate the selection of the appropriate number of clusters (S2 Text). We run the GMM with a range of different values of cluster number K (from 18 to 23) and revealed 22 distinct groups (while K is set to 22, the Davies-Bouldin index reaches the minimum value, S1 Fig).

**Fig 2:**
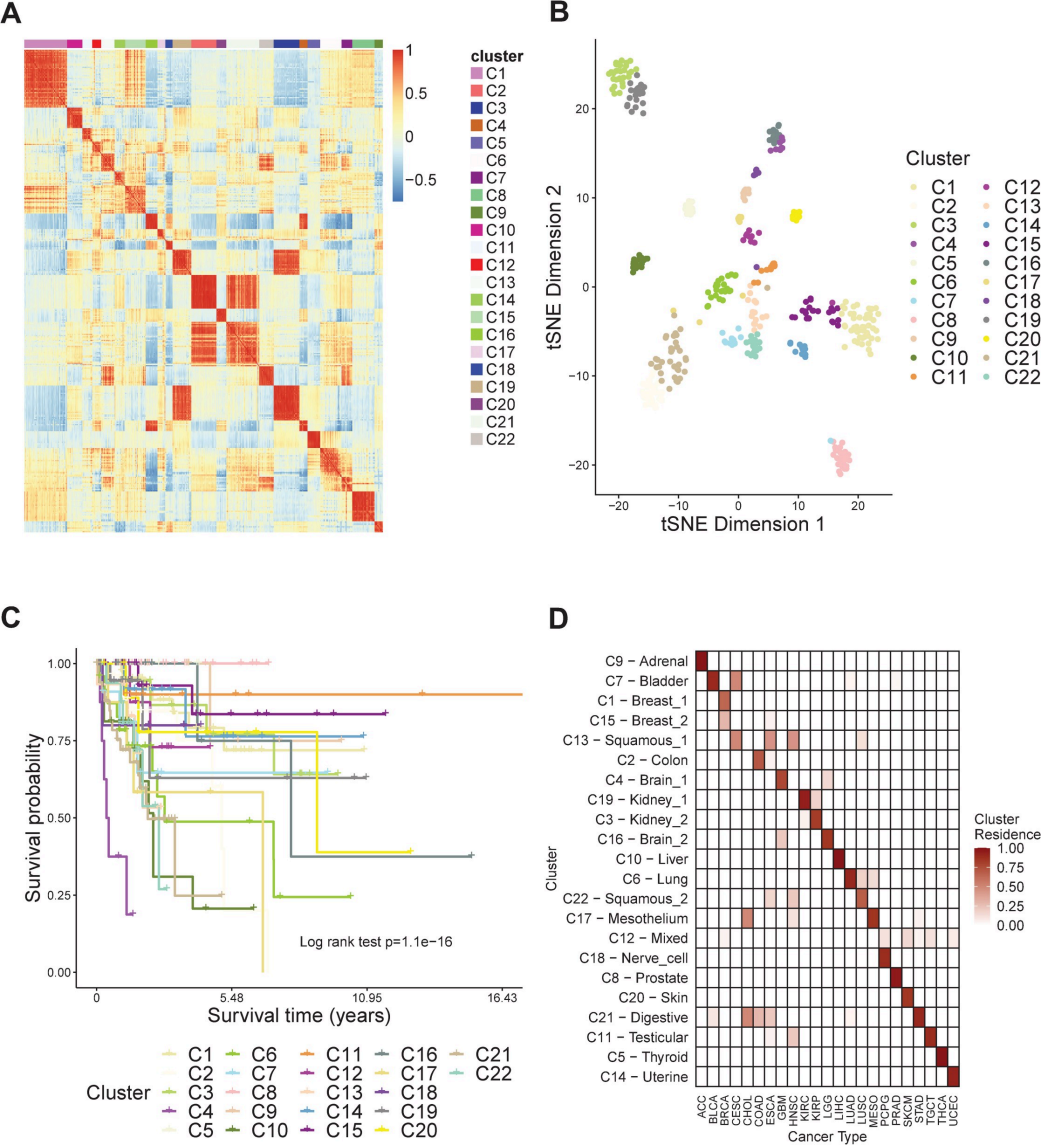
Different patterns of ClusterATAC-clusters in 401 tumor samples from the TCGA. (A) heatmap of the sample similarity matrix showing clear block boundaries. (B) t-SNE visualization on the extracted 200 features from the ClusterATAC. (C) Kaplan Meier survival curve showing that clusters significantly have different survival patterns. (D) heatmap of the cluster residence showing the percent of each cluster that overlaps with each cancer type.

We denoted the 22 clusters as C1-C22 and arranged samples according to the cluster labels. We evaluated the similarities of samples by calculating the correlation of the features generated by Encoder-GAN and performed the heatmap to achieve the visualization of the clusters (Fig 2A). We observed that each identified cluster exhibited a block boundary. Simultaneously, there is a certain correlation between some of the clusters (such as C2 and C21, C3 and C19), which implies that some clusters are related while others are quite different. To have a direct view of the features over clusters C1-C22, we used t-distributed stochastic neighbor embedding [27] (t-SNE) to reduce the vectors from our approach into two dimensions. We visualized the 401 samples with their clustering labels (Fig 2B). Based on the t-SNE map, we observed that samples from the same cluster are always gathered, while samples from different clusters can be distinguished. Furthermore, to demonstrate that there is a significant difference in the survival time of samples corresponding to these different clusters, we performed Kaplan–Meier survival analysis to the overall survival of the 401 TCGA samples (Fig 2C). We found that clusters C1-C22 show significant different survival patterns (log-rank P-value = 1.1e-16). Among all the clusters, C4 (N = 9, dominated by GBM) has the shortest average survival time (712.8 days) while C5 (N = 14, dominated by THCA) and C8 (N = 25, dominated by PRAD) have the longest average survival time (2981.0 days). For all the 22 clusters, over 70% of them are dominated by a single cancer type (C9: ACC; C1, C15: BRCA; C2: COAD; C4: GBM; C19: KIRC; C3: KIRP; C16: LGG; C10: LIHC; C6: LUAD; C17: MESO; C18: PCPG; C8: PRAD; C20: SKCM; C11: TGCT; C5: THCA; C14: UCEC). Most of the remaining clusters contain similar tissue or organ samples (C7, C13, C22: Squamous histology cancers; C21: gastrointestinal cancers). The cluster C12 contains six types of tumors. It exhibits regulatory effects on important cancer-related PI3K genes, suggesting that the molecular subtyping method and histopathology-based method are sometimes different. Based on the above observations, we characterized the cluster labels with cancer cell types (Fig 2D).

Next, we explored the rationality of ClusterATAC's results on the RNA-seq dataset. ClusterATAC achieved five clusters (labeled C1~C5) on the BRCA tumors. We use the heatmap to visualize the similarity matrix of the low-dimensional features of ClusterATAC (S2 Fig). There are block boundaries of all the clusters, while C2, C3, C4, and C5 have a certain degree of correlation. We performed the survival analysis (S3A Fig) on the subtyping results and found that the survival curves of C1 to C5 have significant differences (Log-rank P-value = 4.31e-4). The subtype with the longest average survival time is C1, followed by C5 and C3. The prognosis of the samples in C2 and C4 is poor. Finally, we use TSNE to visualize all the tumors' features and explore the characteristics of each cluster (S3B Fig). Samples belonging to the same subtype are always grouped, while samples belonging to different subtypes are far apart. The unique cluster is C1, which is located on the lower side of the coordinate plane. We also observed that C3 and C5 are in the center of the coordinate plane, while C2 and C4 are located on the coordinate plane's upper side.

### Evaluate the performance of ClusterATAC on benchmark data sets

We used the constructed ATAC-seq data set and RNA-seq data to benchmark clustering methods. On the ATAC-seq data set, according to the previous analysis result [15], we set the clustering number to 18. On the RNA-seq data set, based on the last subtyping results [28], we set the number of clusters to 5. On these data sets, we compared ClusterATAC with four state-of-art clustering algorithms: K-means, spectral clustering (Spectral), autoencoder (AE), variational autoencoder (VAE). K-means and Spectral are the two most stable and effective clustering algorithms, while AE and VAE are two deep learning algorithms applied to omics data clustering [29–31]. Especially, VAE has been proven by previous work to handle high-dimensional ATAC-seq data [20]. On the ATAC-seq data set, to illustrate that the 22-cluster solution of ClusterATAC is also reasonable, we appended ClusterATAC (k = 22) and DensityPeakCluster method [15] for the performance comparison (S3 Text). When classifying tumors, the molecular classification may be more accurate than cancer type (for example, samples belonging to the same cancer type may have different subtypes). Previous studies consider that at least 10% of patients might be classified (and perhaps treated) using molecular classification [12]. Based on the above reasons, we adopted the criteria from the previous study [32] to evaluate the clustering methods' performance: for all the data sets, we reported the p-values of the log-rank test of the clustering results; for the BRCA data set, we also reported the number of significant clinical features (including age, stage, ER status, HER status, tumor's size, number of lymph nodes, metastasis) of each clustering approach.

**Fig 3:**
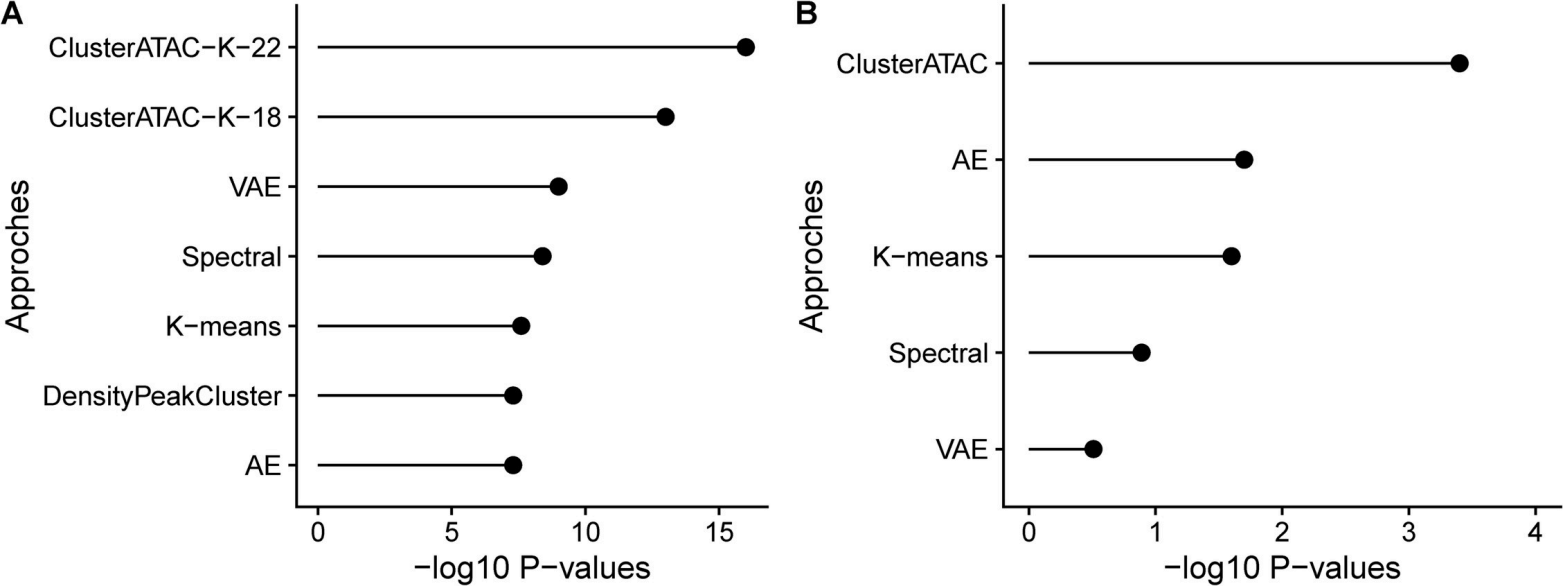
Comparison of ClusterATAC and other clustering methods on A) the ATAC-seq data of 401 samples across 23 tumors. B) the RNA-seq data of 1031 BRCA samples.

On the ATAC-seq data set, each method found the clustering with a significantly different survival (Fig 3, S1 Table). ClusterATAC achieved the best performance (K = 22, P-value = 1.11e-16; K = 18, P-value = 6.71e-14), next was VAE (P-value = 1.06e-9), the third was Spectral (P-value = 3.90e-09), followed by K-means (P-value = 2.76e-08), DensityPeakCluster (4.88e-08), and AE (4.91e-08). On the RNA-seq data set, there were only ClusterATAC, AE, and K-means obtained clustering schemes with significant differences in survival (S2 Table). ClusterATAC achieved the best prognostic value (P-value = 4.31e-04), the second was AE (1.87e-02), the third was K-means (2.51e-02). The survival differences between the clusters obtained by Spectral and VAE are not significant. After the enrichment analyses across the seven clinical parameters, we observed that all methods obtained significant results, while ClusterATAC achieved the smallest p-value in each analysis (S3 Table). Overall, for all the methods, ClusterATAC obtained the most distinct clusters across the two benchmark datasets.

To estimate the performance of different algorithms more accurately, on the two benchmark datasets, we also used permutation tests [32] to calculate the significance of the difference in survival between the clustering solutions and reported the empirical P-values of all the methods across the two benchmark dataset. We found that although the calculation methods of P-values are different, ClusterATAC still outperforms other comparison methods.

### Explore the concordance and difference between clustering schemes

On the ATAC-seq data set, two independent large-scale clustering analyses have been done, and the clustering schemes are found to be highly consistent [12,15]. However, they used utterly different omics data as input. It is essential to see whether the clustering schemes are stable across distinct clustering approaches with the same input data. For this purpose, we focus on exploring the concordance and differences between ClusterATAC and other clustering approaches (DensityPeakCluster, iCluster, K-means clustering, AE, and VAE).

**Fig 4:**
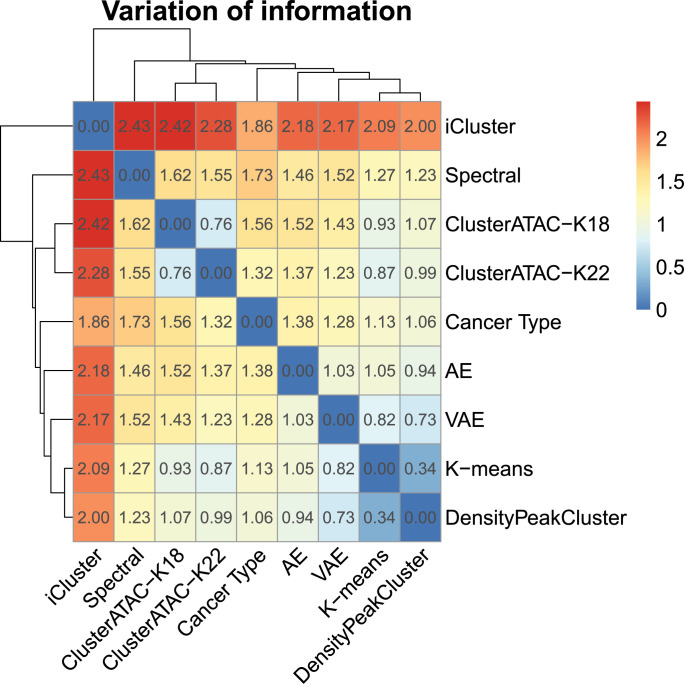
Variation of information analysis of clustering results derived by ClusterATAC and other cancer classification methods (DensityPeakCluster, iCluster, K-means, AE, VAE, and cancer type) on the ATAC-seq data set.

To measure the similarity of distinct clustering methods, we used the variation of information (VI) analysis (Fig 4). Of all the approaches, only iCluster used multi-omics data instead of ATAC-seq data for the clustering, making it the farthest from other methods. The distance matrix suggests that the feature input has more influence on the clustering result than the clustering algorithm. Simultaneously, Fig 4 shows that while using different algorithms, the clustering results of all the methods are stable and consistent with the labels of cancer type (VAE, K-means, and DensityPeakCluster even show a smaller deviation from the cancer type labels than ClusterATAC). The clustering results of ClusterATAC (k = 18) and ClusterATAC (k = 22) indicate that the number of clusters within a reasonable range does not significantly affect the clustering results. It is worth noting that ClusterATAC's clustering scheme is not the same as AE and VAE (Although they all belong to deep learning methods). For example, AE and VAE do not distinguish the brain tumor samples, while both ClusterATAC (k = 18) and ClusterATAC (k = 22) can identify the GBM samples from the LGG samples.

To illustrate that ClusterATAC identified diverse patterns of the TCGA pan-cancers, we analyzed the clustering results of ClusterATAC (C1~C22) and DensityPeakCluster (D1~D18). We found that there are two distinct differences between the results of ClusterATAC and DensityPeakCluster. First, for brain cancer and kidney cancer, the clustering scheme of ClusterATAC is more detailed than the clustering scheme of DensityPeakCluster. The density method classifies all brain cancer patients into one category (D5), while ClusterATAC can more accurately distinguish between GBM (C4) and LGG (C16) in brain cancer. Although both diseases belong to brain cancer, the five-year survival rate of LGG is 59.9%, while GBM is already very serious, and the average survival time of patients is no more than 15 months [33]. Our clustering results indicate that the regulatory elements of the non-coding regions can distinguish GBM and LGG of the brain tissue. The density method classifies all kidney cancers into one category (D1), while ClusterATAC more accurately distinguishes be-tween KIRC (C19) and KIRP (C3) in kidney cancer. Both KIRC and KIRP are histological clusters of renal cell carcinoma (RCC), but KIRP patients are more likely to experience significantly worse clinical outcomes [34]. Distinguish KIRC and KIRP from the perspective of ATAC peak data will help to understand how the non-coding regions contribute to the prognosis assessment of them. Moreover, ClusterATAC has a heterogeneous cluster (C12), and the DensityPeakCluster does not get a mixed category. We found that in the ClusterATAC clusters, almost all patients with C12 correspond to the D17 (mesothelium) of density clusters. Patients with D17 are concentrated in C17 and C12 (S4 Fig). However, C12 does not contain any patients with MESO, and the patients of MESO are mainly in the C17. The cluster C12 contains six different cancer categories, which are significantly different from C17. All of this makes it reasonable to separate C12 and C17 from the same cluster.

Besides, we analyzed the clustering results of ClusterATAC with the 28-iClusters solution [12]. Although the cancer cell types influence the clustering results of the two methods, most of the heterogeneous clusters of ClusterATAC (C7, C12, C13, C21, C22) are entirely different from the previous 28-cluster solution. More interestingly, the ClusterATAC-C12 and iCluster-I20 are both mixed clusters, but they do not have any similarities (S5 Fig). These results illustrate that cancer classification based on ATAC peak data also followed the cell-of-origin patterns. Moreover, the deep learning approach can also find some heterogeneous clusters that correspond to multiple cancer types. The heterogeneous clusters found by different methods are challenging to map to each other.

### Identify cluster-specific loci and genes of tumors

The clustering results of ClusterATAC suggested that different types of cancer have different biomarkers in the non-coding regions, and some biomarkers of the mixed clusters are associated with multiple types of tumors. Based on the clustering solution of ClusterATAC, we explored the biomarkers corresponding to different clusters across the whole genome, obtained their corresponding genes, and analyzed their regulatory mechanisms. For each of the 22 clusters, we marked the target cluster samples with positive labels, and the other individuals are labeled as negative labels. We used the random forest as the feature selection algorithm to identify its corresponding biomarkers. The random forest method input is the ATAC peaks of the samples, and the output is the 0 or 1 labels. The Gini importance of the random forest is used for the selection of candidate biomarkers. After the model fitting, we reported the five most critical non-coding regions and their associated genes for each cluster (S4 Table).

Based on these findings, we conducted a literature search to obtain reliable cluster-specific biomarkers (Table 1). Firstly, we investigated the clusters that follow the "cell-of-origin patterns" (C1~ C6, C8~C11, C14~C18, C20). For the C1 (BRCA), the most critical region is at 8q22.1. Copy number enhancement occurring in this locus is thought to increase the likelihood of metastatic recurrence of breast cancer [35]. At the same time, the linked gene of this region is NDUFAF6, which acts as the coactivator and facilitator of TP53 activity [36]. The 1p22.3 locus is also critical for C1. The variant rs12118297 in this region has been found to increase the risk of breast cancer [37]. For the C2 (COAD), the representative region is at 13q14.3. The previous study showed that chromosomal alterations in this region could significantly affect the survival time of colorectal cancer patients [38]. The 2q33.1 locus is also related to C2. The lower expression of SATB2 is correlated with the clinical diagnoses and the recurrence rate of the colorectal tumor [39]. For the C3 (KIRP), the most representative region is at 13p14.1, and its corresponding gene is ADAMTS9. Recent studies showed that the lncRNA ENSG00000241684 is closely related to clear cell renal cell carcinoma (CCRCC) prognosis [40]. The essential region of C4 (GBM) is 18q23, which is the risk loci of GBM [41]. Among the genes corresponding to the Top5 critical regions of C5 (THCA), the TG gene is found to be closely related to thyroid cancer. Statistical analysis indicated that somatic mutations that occurred on the TG gene were associated with a poor clinical outcome in patients with thyroid cancer [42]. SFTPB is one of the representative genes for C6 (LUAD). A recent study suggested using the expression of SFTPB as a prognostic marker for lung cancer patients [43]. The most critical region of C8 (PRAD) is 11p15.4, which is proved to be a susceptibility locus for prostate cancer [44]. The representative region of C9 (ACC) is 19q13.33, and previous studies showed that copy number aberrations in this locus directly lead to the poor survival of adrenal [45]. The representative gene of C10 (LIHC) is ORM1, which is considered as a prognostic biomarker for hepatocellular carcinoma [46]. The most representative gene of C11 (TGCT) is TET1 (regulated by 10q21.3). Previous work showed that in some TGCT samples, methylation of TET1 deregulated [47]. The representative locus of C14 (UCEC) is 1q32.1. Previous work showed that somatic copy number amplifications occurring in this locus lead to poor prognosis of endometrial cancers [48]. C15 is another subgroup of BRCA, and the most representative gene corresponding to it is IRX5 (regulated by 16q12.2). Experiments showed that knocking down the IRX5 gene in the breast cancer cell leads to a decrease in cell survival [49]. Three representative regions of C16 (LGG) were located at 1q22, and one of them was linked to the BCAN gene, which is considered a central factor in promoting glioma progression [50]. Three representative regions of C17 (MESO) were located at 15q22.2 and regulate BNC1. The study found that Epigenetic alterations occurred in the BNC1 gene in the mesothelioma cell line and may be involved in mesothelioma progression [51]. Two representative regions of C18 (PCPG) were located at 9q34.2, and both of them regulated the DBH gene, suggesting a correlation between the DBH gene and PCPG. Indeed, DBH had been used as a marker to identify PCPG [52]. GPX3 is one of the representative genes of C19 (KIRC). There is already evidence that the expression of GPX3 is significantly downregulated in primary renal tumors [53]. For C20 (SKCM), the most representative gene is TRPM1 (regulated by 15q13.3), which is considered to be a metastasis-related important gene of skin cancer. The expression of TRPM1 is directly related to the clinical prognosis of SKCM patients [54].

**Table 1:** The representative cluster-specific non-coding loci (chromosome, start, end) and their linked genes and the Gini Importance

Subgroup	Chromosome	Start	End	Linked Gene	Gini Importance
C1	chr8	94405202	94405703	NDUFAF6	0.01781
	chr1	85215748	85216249	SYDE2	0.00941
C2	chr13	52155104	52155605	NEK3	0.01460
	chr2	199942283	199942784	SATB2	0.00966
C3	chr13	48154309	48154810	ITM2B	0.01684
C4	chr18	77521941	77522442	GALR1	0.01920
C5	chr8	132933462	132933963	TG	0.01000
C6	chr2	85660680	85661181	SFTPB	0.00704
C7	chr10	47483847	47484348	ANXA8	0.00873
C8	chr11	4636027	4636528	OR51E1	0.01726
C9	chr19	49790844	49791345	SIGLEC11	0.01000
C10	chr9	114078164	114078665	ORM1	0.02000
C11	chr10	68553162	68553663	TET1	0.01000
C12	chr10	96642909	96643410	PIK3AP1	0.00597
C13	chr1	3473515	3474016	TP73	0.00716
C14	chr1	206061893	206062394	C1orf186	0.00274
C15	chr16	54647149	54647650	IRX5	0.00662
C16	chr1	156422205	156422706	BCAN	0.01000
C17	chr15	83286947	83287448	BNC1	0.01000
C18	chr9	133627172	133627673	DBH	0.01000
C19	chr5	151014839	151015340	GPX3	0.00606
C20	chr15	31102405	31102906	TRPM1	0.01000
C21	chr20	22619200	22619701	FOXA2	0.00715
C22	chr18	63586359	63586860	SERPINB5	0.00736

Next, we explored the heterogeneous clusters and their corresponding critical loci. For squamous histology cancers, three clusters were corresponding to them: C7, C13, C22. Among them, the representative region of C7 corresponds to the gene ANXA8. Studies have shown that ANXA8 is a molecular marker associated with lymph node metastasis in oral squamous cell carcinoma [55]. The representative gene of C13 is TP73 (p53 family of transcription factors), is a tumor suppressor. TP73 is considered to be associated with head and neck squamous cell carcinoma [56]. SerpinB5 is one of the relevant regulatory genes of C22. The expression of SerpinB5 is significantly down-regulated in patients with esophageal squamous cell carcinoma [57]. C21 is responsible for gastrointestinal cancers. The representative gene of C21 is FOXA2, which is a suppressor in a wide range of tumors. For gastric cancer, the clinical prognosis of patients is related to the expression of FOXA2 [58]. PIK3AP1 (one of the PI3K pathway genes) is one of the regulatory genes of the mixed cluster C12. Since the PI3K pathway is one of the most common signaling pathways of cancer, among all the clusters, C12 contained the most types of cancer patients. Above all, for each cluster, we found that there were limited regulatory genes in the cancer signaling pathways (only the mixed cluster C12 associated with the PI3K pathway). The representative loci and genes of different clusters are always distinct.

## Discussion

Currently, deep learning methods are gradually being used for the analysis of cancer genomic data. In this work, we proposed ClusterATAC, a deep-learning-based clustering model for the cancer classification. The central component of ClusterATAC is the Encoder-GAN, which can learn the nonlinear representation of the complex raw data and transfer them to the coded low dimensional features. With these extracted features, GMM is another component responsible for the unsupervised clustering. Moreover, we used the Davies-Bouldin index to determine the appropriate number of clusters from a reasonable range of values. ClusterATAC successfully obtained 22 clusters form the ATAC-seq profiles of 401 TCGA samples. We observed that most of the clusters follow the ‘Cell-of-Origin’ pattern, which is consistent with the recent study. There were significant survival differences between the 22 clusters. More than 70% of the clusters were homogeneous for a single cancer type. On the ATAC-seq dataset and RNA-seq dataset, ClusterATAC has achieved excellent performance. We used the random forest to select the representative loci and the corresponding regulatory genes on each cluster of the Pan-cancer data set. These loci and genes are always tumor-specific and responsible for the occurrence and development of the related tumor. These findings indicated that the ClusterATAC clustering results have the potential opportunity to develop cancer therapeutics.

The 22-cluster solution of ClusterATAC reveals the critical role of regulatory elements in non-coding regions for cancer classification. The input of the model is the ATAC peak score of the non-coding loci so that each cluster can finally link to representative non-coding loci and regulatory genes. Since most of the clusters link to a specific tumor type, we can indicate that a significant number of genomic loci and regulatory genes are tumor-specific. For example, C5 refers to thyroid cancer, whose representative locus is 8q24.2, and the regulatory gene is TG. They are closely related to thyroid cancer since the corresponding protein of the TG gene is produced by the thyroid gland. For another example, the representative region of C16 is 1q22, and its regulatory gene BCAN is essential in promoting the progression of glioma.

An essential component of ClusterATAC is Encoder-GAN, a model based on the generative adversarial network. Recently, GAN architecture has become the most popular generation model. To the best of our knowledge, ClusterATAC is the first to introduce GAN for the modeling of ATAC-seq data. The GAN architecture has many extensions and improvements, depending on the scenario of applications. Most GAN-based models focus on sampling from random distributions and generating high-quality samples. These models combine the discriminator with the generator and improve the quality of the generated data. In contrast, Encoder-GAN introduces adversarial learning of the discriminator and the encoder and performs a nonlinear representation of raw data accurately. At the same time, we downgrade the decoder network structure and minimize the reconstruction error to maximize the description of the encoder in the autoencoder architecture. These innovative network designs enhance the representation ability of the approach.

ClusterATAC has limitations. Currently, it only supports the clustering of single omics data. Since most methods of molecular classification are based on the multi-omics data integration, to obtain more accurate clustering results, our future work is to collect somatic mutation, gene expression, DNA methylation, protein expression, and other omics data, and introduce GAN into integrative clustering analysis to expect more meaningful results.

## Methods

### Overview of ClusterATAC

The ClusterATAC framework took the genome-wide high-dimensional omics data as the input and predicted the cluster labels of each sample across the 23 TCGA cancer types. The first component of the ClusterATAC framework is Encoder-GAN, which reduces features to low-dimensional space for running clustering algorithms. In deep learning, autoencoder is frequently used for nonlinear dimensionality reduction in an unsupervised manner. Autoencoder is composed of multiple coding layers (encoder) and decoding layers (decoder). The encoder is corresponding to the learning of efficient nonlinear data representation. Similar to the PCA, the encoder provides a low-dimensional representation of sophisticated features. The decoder maps the low dimensional space to the original data space. The combination of Encoder and Decoder completes the reconstruction of the data. We proposed Encoder-GAN as a Deep architecture based on the WAE framework [59]. It accurately represented nonlinear high-dimensional input features by solving the min-max problem between two adversarial networks. The second component of ClusterATAC is GMM-clustering, which uses the latent variables from the Encoder-GAN as the input. The probabilistic model is based on GMM and focuses on discovering different patterns across different cancers. Based on the identified subgroups, we can also obtain the class labels corresponding to each sample.

### Dimensionality reduction using Encoder-GAN

Encoder-GAN was developed based on autoencoder architecture. It made two improvements. Firstly, since the goal is to learn the accurate representation of the raw data and then perform robust cluster analysis, but not to reconstruct the raw data, the decoder of Encoder-GAN responsible for data generation is simplified to the linear regression model. By downgrading the decoder structure and minimizing the reconstruction error, the representation capability of the encoder achieved maximum enhancement. Secondly, we introduced a discriminator to the encoder of the network (different from the design of most GANs which enhance the ability of the decoder). In the related research of GAN, the min-max game is usually between discriminator and decoder to strengthen the decoder's ability to generate samples from the random distribution. However, since our model does not need the data generation, but aim to improve the low-dimensional representation with the encoder, inspired by the idea of the WAE framework, we lead a min-max game between discriminator and encoder. The role of the discriminator in Encoder-GAN is to distinguish whether the latent distribution matches the prior. Encoder-GAN emphasizes that the latent variable's distribution should close to the prior, thereby improving the accuracy of the coding network.

The encoder takes the input signal of **x** and transfers them to the latent representation **z.** At the same time, the decoder uses the input of **z** and transfer it to the reconstructed signal **x**':

$$\begin{array}{l} z\sim Q\left(z|x\right)\\ x'\sim G\left(x'|z\right) \end{array}$$ (1)

Where *Q*(**z**|**x**) is the density function of the encoder, and *G*(**x**'|**z**) is the decoder's density function. We used the squared loss *L*_REC_ to minimize the Euclidean distance between **x** and **x**' and enhance the representation performance of the encoder:

$$L_{REC}=\frac{1}{n}\sum_{i=1}^{n}{\left\|x-x'\right\|}_{2}^{2}$$ (2)

Next, we structured the latent variable **z** and assumed that it follows to the prior distribution *P*(**z**). Let *D*() be the discriminator function, **z**' be the positive points that sampled from the *P*(**z**), and z be the negative points from the output of encoder *Q*(z|x). The discriminator is trained with encoder together to distinguish **z** and **z**'. We used the min-max game-based adversarial training to update the parameters of encoder Q and discriminator D simultaneously:

$$\underset{Q}{\min}\;\underset{D}{\max}E_{z'∼P\left(z\right)}\left(\log\left(D\left(z'\right)\right)\right)+E_{z∼Q\left(z|x\right)}\left(\log\left(1-D\left(z\right)\right)\right)$$ (3)

The discriminator D is learned to distinguish the samples from the prior distribution *P*(z) and the encoder outputs from the posterior distribution *Q*(**z**|**x**). The encoder Q is learned to make the encoded output as close as possible to the prior *P*(z). With the adversarial learning, the performance of the encoder and the discriminator was improved. We used the loss functions of the two networks to facilitate the solution using the gradient algorithm and combined them as the loss function of the GAN. This training process aims to minimize adversarial loss:

$$\begin{array}{l} L_{D}=-E_{z'∼P\left(z\right)}\left(\log\left(D\left(z'\right)\right)\right)-E_{z∼Q\left(z|x\right)}\left(\log\left(1-D\left(z\right)\right)\right)\\ L_{G}=-E_{z∼Q\left(z|x\right)}\left(\log\left(D\left(z\right)\right)\right)\\ L_{GAN}=L_{D}+L_{G} \end{array}$$ (4)

Where *L*_D_ is the loss of the discriminator, *L*_G_ is the loss of the decoder, and *L*_GAN_ is the loss of the GAN. Next, the GAN process is combined with the reconstruction process:

$$L_{ALL}=\lambda_{1}L_{GAN}+\lambda_{2}L_{REC}$$ (5)

Where λ_1_ is the weight of *L*_GAN_, and λ_2_ is the weight of *L*_REC_. For each iteration of the training. The model parameters of the discriminator are updated based on *L*_D_ by descending:

$$\frac{1}{n}\sum_{i=1}^{n}\lambda_{1}\left(-\log D\left(z_{i}\right)-\log\left(1-D\left(z{'}_{i}\right)\right)\right)$$ (6)

Since the encoder network participates in the two modules: reconstruction and prior regularization, we combined *L*_*GAN*_ and *L*_*REC*_ to update the model parameters of Q and G by descending:

$$\frac{1}{n}\sum_{i=1}^{n}\left(-\lambda_{1}\log D\left(z{'}_{i}\right)+\lambda_{2}{\left\|x_{i}-G\left(z{'}_{i}\right)\right\|}_{2}^{2}\right)$$ (7)

The network structure of Q, G, and D are shown in S5 Table. During the model training process, the three networks' parameters are updated in turn until the model achieves converge (S1 Text). After the model fitting is completed, we took all the ATAC-peak data as input and used the encoder's output as the input of the GMM clustering component.

### Clustering using Gaussian mixture model

We used the latent factors extracted from the Encoder-GAN as the input for the clustering component of our framework. The clustering procedure is based on the Gaussian Mixture Model (GMM). The model can be thought of as a generalized form of the k-means clustering. Relative to the hard decision of the K-means, it supports to generate the probability that each patient belongs to a different subgroup. Let $H={\left\{h_{n}\right\}}_{n=1}^{N}$ be the input matrix of the latent space of the original ATAC-peak data, where N is the number of samples. The model describes the latent space **H** with a mixture of finite Gaussian distributions. For the patient with index n, the feature input for the model is denoted as **h**_n_. Let M be the number of mixture components, and *p*_i_() be the density function of the ith Gaussian distribution. The density function of the model takes the form:

$$p\left(h_{n}\right)=\sum_{i=1}^{M}\pi_{i}p_{i}\left(h_{n}\right)=\sum_{i=1}^{M}\pi_{i}N\left(h_{n}|\mu_{i},\Sigma_{i}\right)$$ (8)

Where **μ** and **Σ** are the mean and covariance of the Gaussian distributions, **π** = (π_1_, π_2_,…, π_M_) is the weight of the Gaussian component of the model. In the training process of the model, the parameters $\theta ={\left\{\pi_{i},\mu_{i},\Sigma_{i}\right\}}_{i=1,\ldots ,M}$ need to be updated. We used the EM algorithm to update the parameters with the training data (S2 Text). After the model training is completed, the cluster labels of the samples can be predicted based on calculating the posterior probabilities of the different clusters.

### Compilation of the data set

We collected ATAC-seq data and RNA-seq data from the TCGA project. The ATAC-seq data set includes 23 types of cancer of the TCGA Pan-Cancer Atlas[12] (ACC, BLCA, BRCA, CESC, CHOL, COAD, ESCA, GBM, HNSC, KIRC, KIRP, LGG, LIHC, LUAD, LUSC, MESO, PCPG, PRAD, SKCM, STAD, TGCT, THCA, UCEC). The RNA-seq data set includes all the TCGA BRCA tumors. The clinical information (such as ‘vital status’, ‘days to death’, ‘days to last followup’) are used to evaluate the clustering results. To analyze these results more comprehensively, we collected iCluster analysis results (28 clusters) and DensityPeakCluster results (18 clusters) from previous studies [12,15].

## Supporting information

S1 Fig Summary of the training process of the Encoder-GAN Model. A) The change of the discriminative loss and generative loss of GAN in each cluster during the training process. B) The change of the Davis-Bouldin index during the training process.
(TIF)

S2 Fig Heatmap of the correlation matrix of the ClusterATAC low-dimensional features on the 1031 TCGA BRCA data set. (TIF)

S3 Fig Different patterns of ClusterATAC-clusters on the TCGA BRCA data set. (A) Kaplan Meier survival plot showing that clusters significantly have different survival patterns. (B) t-SNE visualization on the extracted 200 features from the model.
(TIF)

S4 Fig Heatmap of the cluster residence shows the percent of ClusterATAC clusters (C1~C22) that overlap with the density clusters of DensityPeakCluster (D1~D18). (TIF)

S5 Fig Heatmap of the cluster residence shows the percent of each ClusterATAC-cluster (C1~C22) that overlaps with each iCluster (I1~I28). (TIF)

S1 Table Benchmark results of the clustering methods on the ATAC-seq dataset. (XLSX)

S2 Table Performance comparison of ClusterATAC and other algorithms on the RNA-seq dataset. (XLSX)

S3 Table Enrichment tests for the clinical features of ClusterATAC and other algorithms on the RNA-seq dataset. (XLSX)

S4 Table The representative non-coding loci (chromosome, start, end) and their linked genes of the 22 clusters. (XLSX)

S5 Table Deep architectures of ClusterATAC, autoencoder, and variational autoencoder. (XLSX)

S1 Text The model training of ClusterATAC. (DOCX)

S2 Text Details of the comparison of the clustering approaches. (DOCX)

S3 Text The implementation details of GMM and random forest. (DOCX)
