# Supplementary figures and images for: Cancer classification based on chromatin accessibility profiles with deep adversarial learning model
Source: PLoS Comput Biol. 2020 Nov 9;16(11):e1008405. doi: 10.1371/journal.pcbi.1008405 (PMC7676699; doi:10.1371/journal.pcbi.1008405)

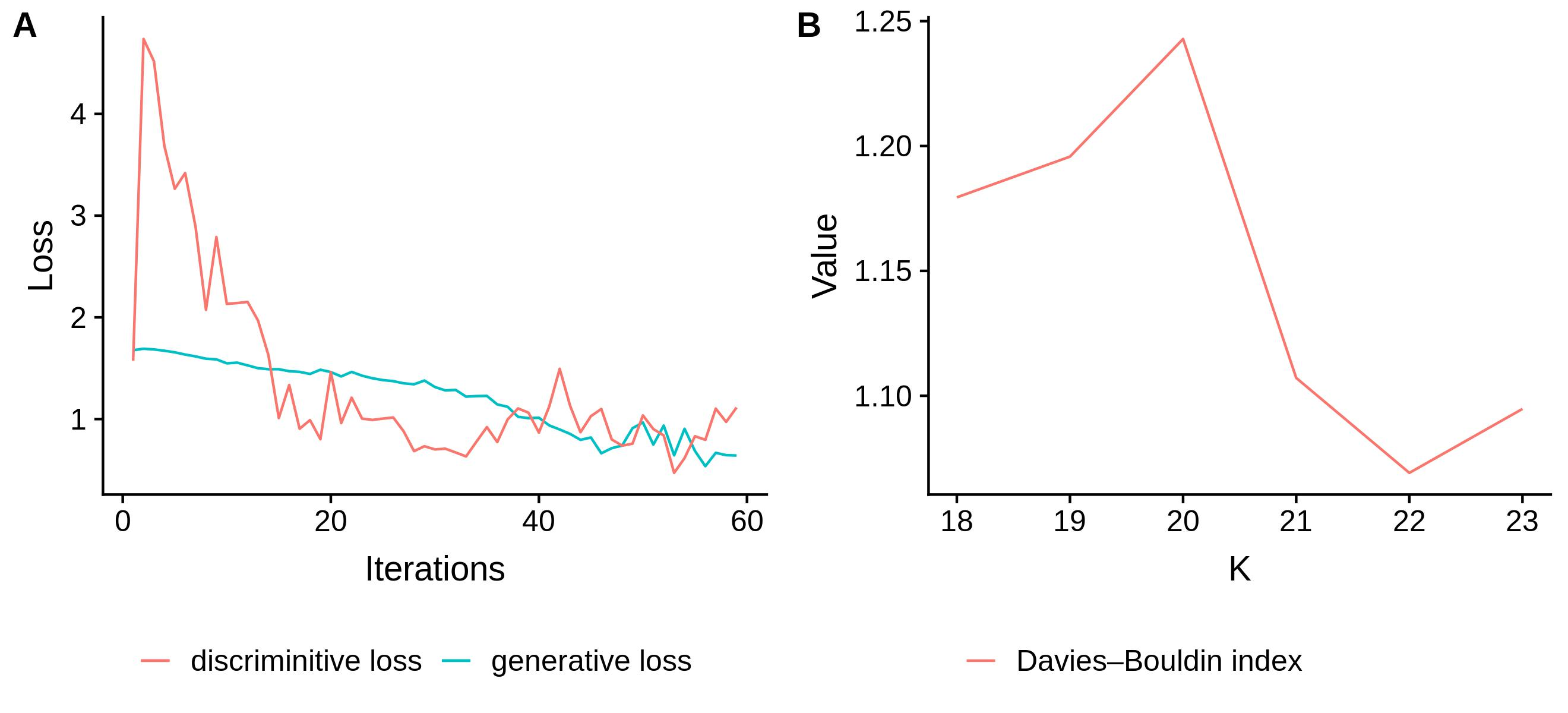

Supplement: S1 Fig — A) The change of the discriminative loss and generative loss of GAN in each cluster during the training process. B) The change of the Davis-Bouldin index during the training process. (TIF) [file pcbi.1008405.s001.tif]

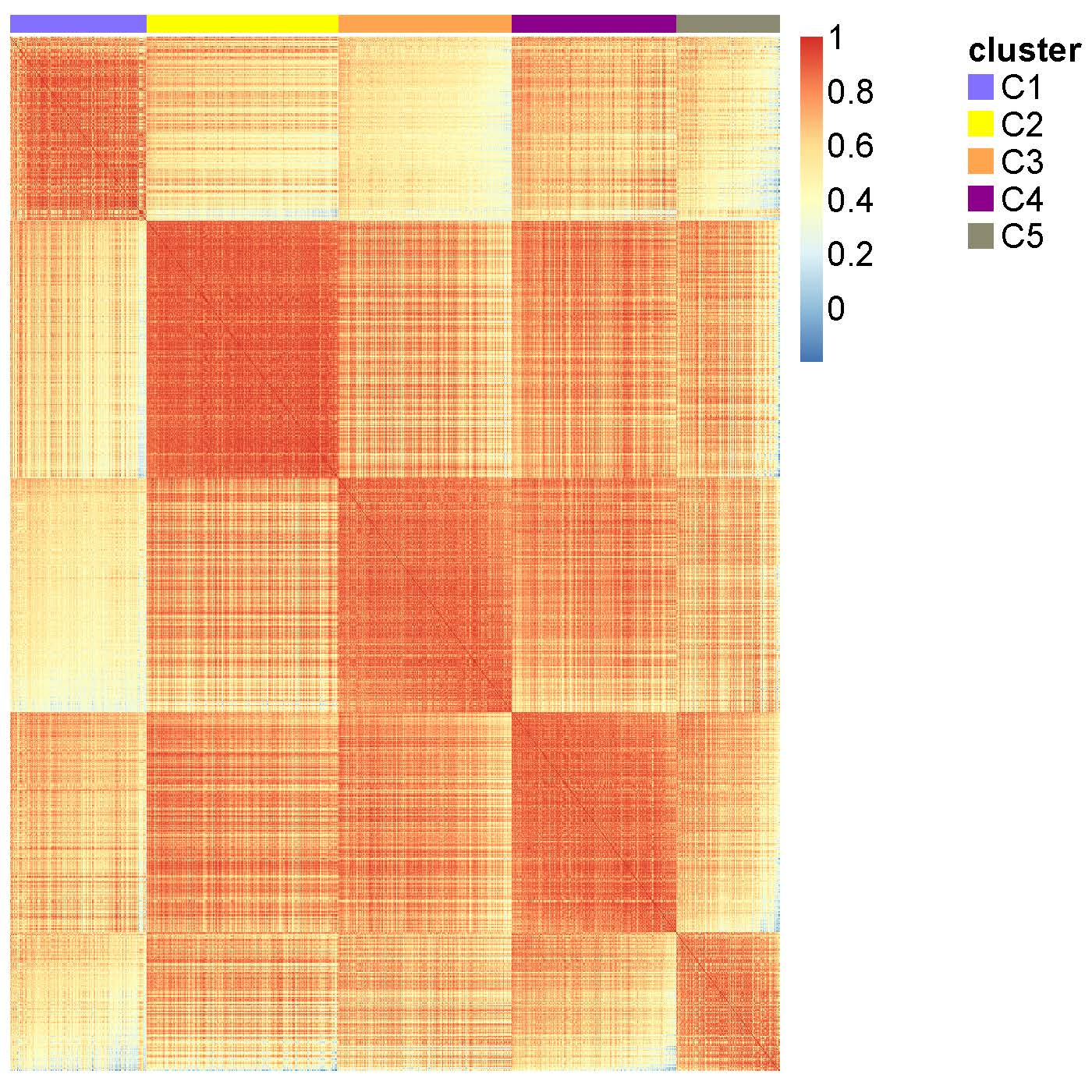

Supplement: S2 Fig — (TIF) [file pcbi.1008405.s002.tif]

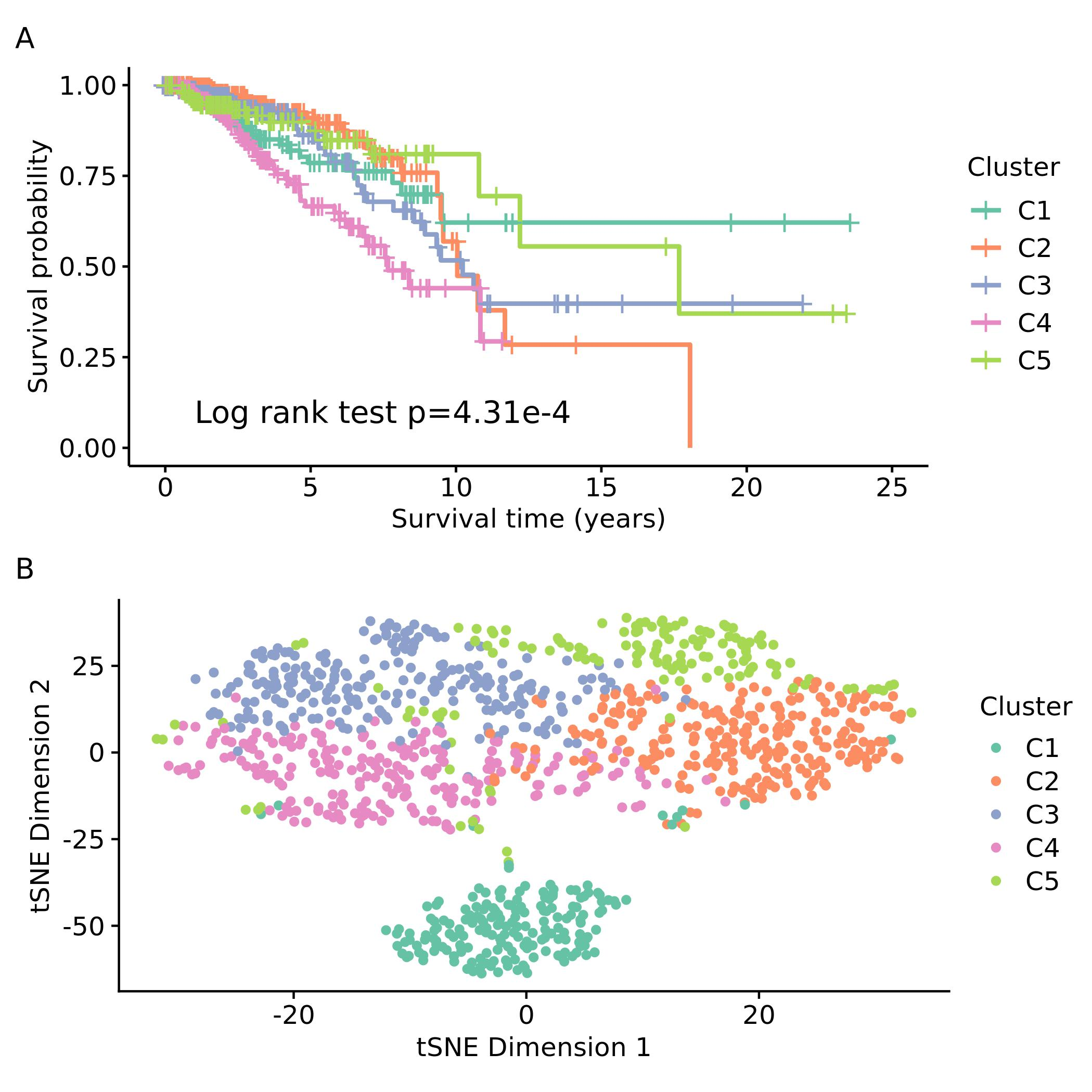

Supplement: S3 Fig — (A) Kaplan Meier survival plot showing that clusters significantly have different survival patterns. (B) t-SNE visualization on the extracted 200 features from the model. (TIF) [file pcbi.1008405.s003.tif]

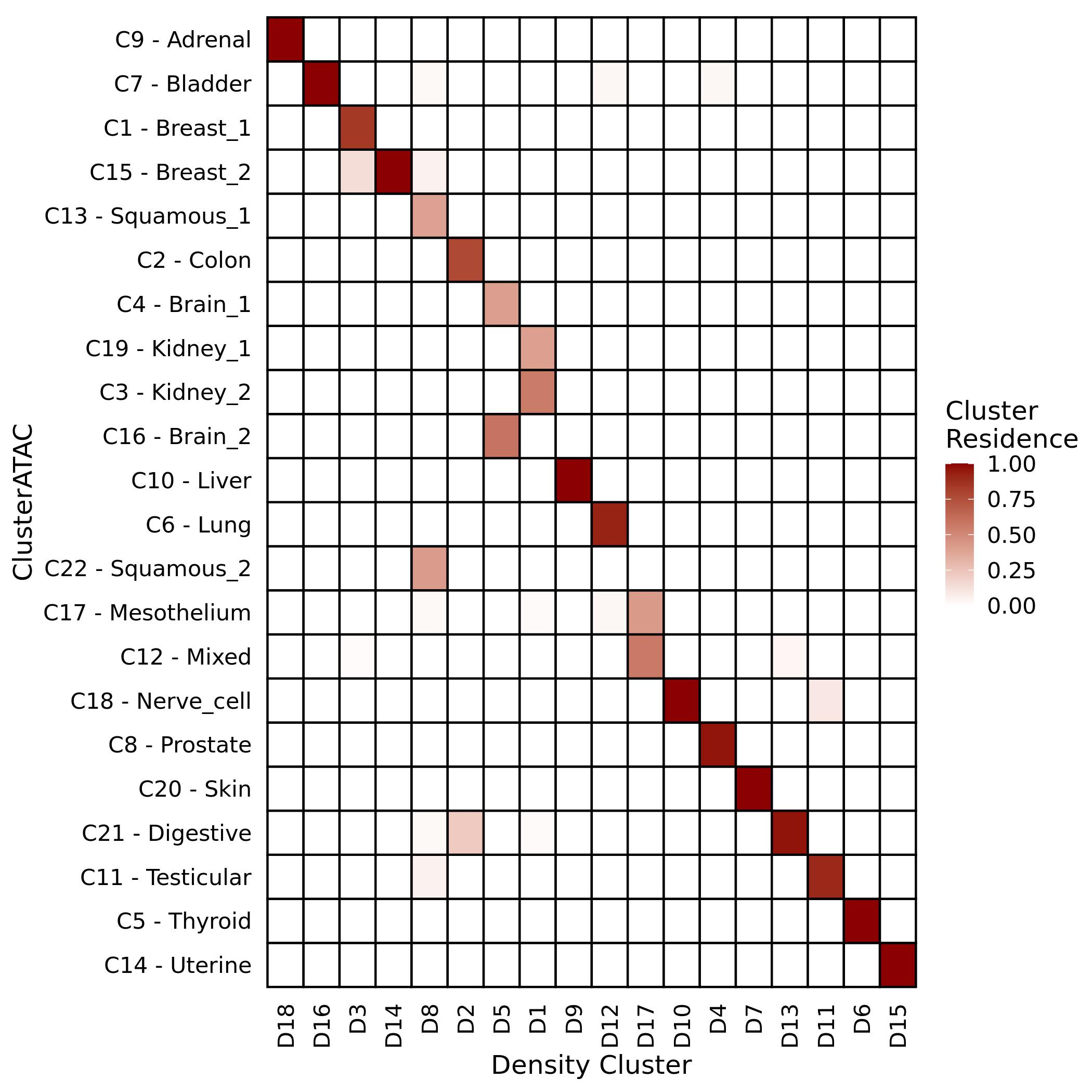

Supplement: S4 Fig — (TIF) [file pcbi.1008405.s004.tif]

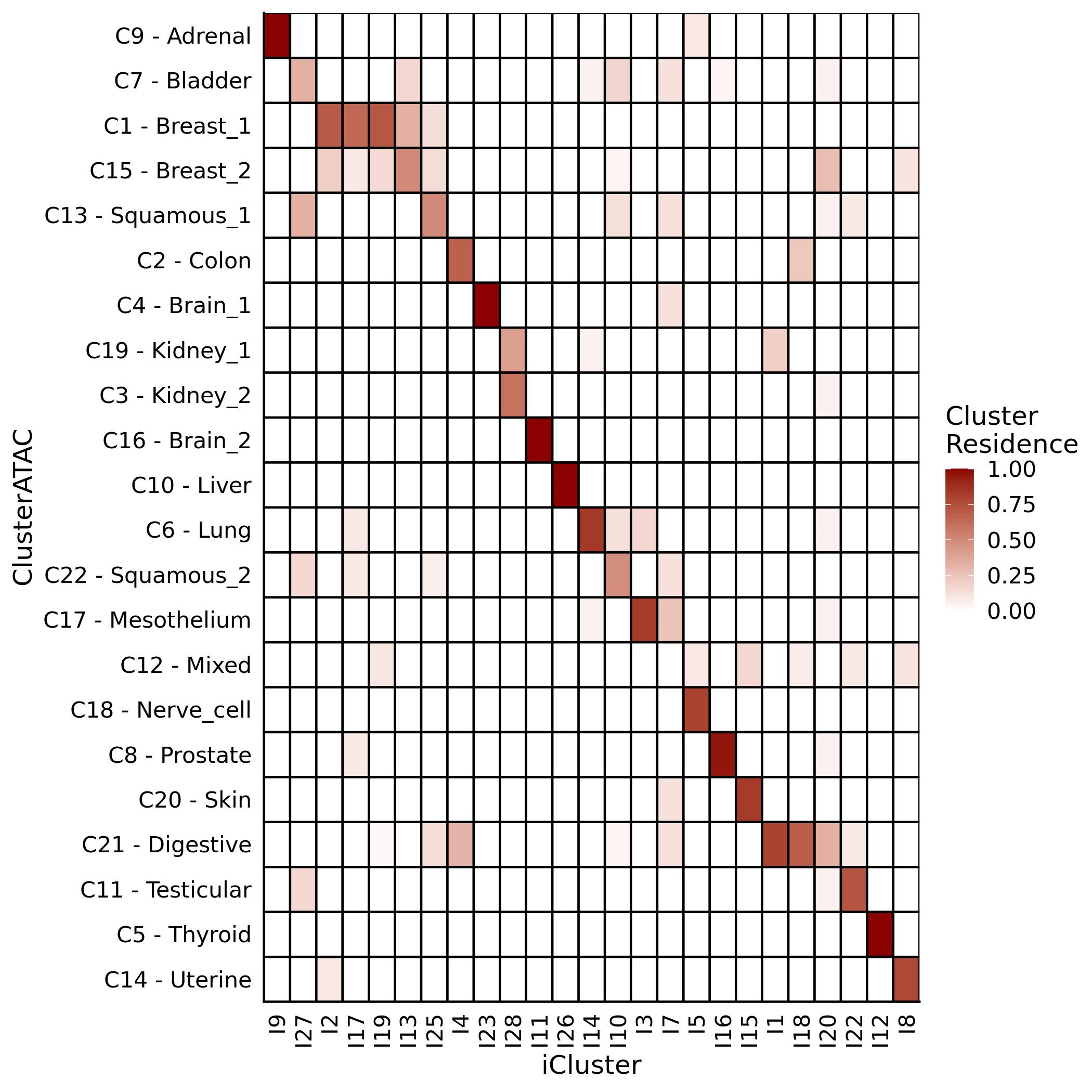

Supplement: S5 Fig — (TIF) [file pcbi.1008405.s005.tif]
