# Supplementary material for: Cancer classification based on chromatin accessibility profiles with deep adversarial learning model
Source: PLoS Comput Biol. 2020 Nov 9;16(11):e1008405. doi: 10.1371/journal.pcbi.1008405 (PMC7676699; doi:10.1371/journal.pcbi.1008405)
Supplement: S1 Text — (DOCX) [file pcbi.1008405.s011.docx]

## S1 Text: The model training of ClusterATAC

We proposed ClusterATAC based on the generative adversarial network (GAN) architecture [1, 2]. The traditional GAN focuses on generating new high-quality data, so its discriminator is often used to enhance the performance of the generative network. However, cancer classification does not need new data generation.

We combined the discriminator with the encoder for adversarial training to improve the representation power of the encoder with the limited but high-dimensional input data. Second, for the same propose, since there is no need to generate new data, we are most likely to simplify the generative network and downgrade the decoder to the linear regression model. Finally, the model parameters of the encoder are optimized in the process of minimizing the reconstruction error. In the model training process, since there is a min-max game between the discriminator and the encoder, we use the early-stop mechanism to prevent model over-fitting. When the ideal model stops training, the features automatically extracted by the encoder are followed the prior assumption of the input. The discriminator cannot distinguish it from the data sampled from the a priori distribution, so the accuracy of the discriminator should be as close as possible to 50%. At this time, the loss of the encoder also falls to a stable range, at which point we stop the model training and save the training parameters of the networks.

For the ATAC-seq data from 401 people in this study, we recorded the changes in the training process's loss. In keeping with the generic version of GAN, here we used the generative loss to represent the loss of the encoder, and discriminative loss to describe the loss of the discriminator. Since our framework focuses more on reducing the reconstruction error than the discriminator error, following the strategy described in the super-resolution GAN [3], for all the data set, we set λ_1_=1.00e-3, λ_2_=9.99e-1. For the ATAC-seq data after 20 iterations, the training of the encoder and discriminator is stabilized (S1 Fig). When the model training reached the 59th iteration, the accuracy of the discriminator reaches 49.9%, which is very close to 50%. At this time, the early-stop mechanism is triggered, then the model training is completed.

Reference

1. Gupta AZ, James. Feedback GAN for DNA optimizes protein functions. Nature Machine Intelligence. 2019;1(2):105.

2. Goodfellow IJ, Pouget-Abadie J, Mirza M, Xu B, Warde-Farley D, Ozair S, et al. Generative Adversarial Nets. Adv Neur In. 2014;27. PubMed PMID: WOS:000452647101094.

3. Ledig C, Theis L, Huszar F, Caballero J, Cunningham A, Acosta A, et al. Photo-Realistic Single Image Super-Resolution Using a Generative Adversarial Network. 30th Ieee Conference on Computer Vision and Pattern Recognition (Cvpr 2017). 2017:105-14. doi: 10.1109/Cvpr.2017.19. PubMed PMID: WOS:000418371400012.
