# Supplementary material for: Cancer classification based on chromatin accessibility profiles with deep adversarial learning model
Source: PLoS Comput Biol. 2020 Nov 9;16(11):e1008405. doi: 10.1371/journal.pcbi.1008405 (PMC7676699; doi:10.1371/journal.pcbi.1008405)
Supplement: S2 Text — (DOCX) [file pcbi.1008405.s012.docx]

## S2 Text: Details of the comparison of the clustering approaches

We have compared ClusterATAC with the state-of-art clustering methods (such as K-means, Spectral, AE, VAE) on the ATAC-seq dataset and RNA-seq dataset. Moreover, we also showed the performance of DensityPeakCluster [1] on the ATAC-seq dataset [2]. Since the inputs on both benchmark datasets are high-dimensional omics data, to avoid the over-fitting of the model, we used the PCA technique before the K-means algorithm and the Spectral clustering algorithm. The PCA, GMM, K-means, Spectral methods are all implemented with scikit-learn (version 0.21.3).

The variation of information analysis was done using the ‘mcclust’ package (1.0) of R (version 3.6.1). In terms of implementation, ClusterATAC, AE, and VAE keep the network layer consistent as much as possible (see Table S5). Moreover, while the model is trained, the number of iterations and the loss function of all methods use the loss function recommended by the relevant papers. The optimization function for all methods is the Adam function.

All approaches were run on the same machine using the same runtime. The implementation of the neural networks was based on the open-source Python library Keras 2.2.4 and Tensorflow 1.15.0 (GPU version). The operating system is Ubuntu Linux release 18.04. The CPU is Intel Xeon E5-2695 v2, and the GPU is NVIDIA TITAN XP.

Reference

1. Rodriguez A, Laio A. Clustering by fast search and find of density peaks. Science. 2014;344(6191):1492-6. Epub 2014/06/28. doi: 10.1126/science.1242072. PubMed PMID: 24970081.

2. Corces MR, Granja JM, Shams S, Louie BH, Seoane JA, Zhou WD, et al. The chromatin accessibility landscape of primary human cancers. Science. 2018;362(6413):420-33. doi: 10.1126/science.aav1898. PubMed PMID: WOS:000450441900039.
