# Supplementary material for: Cancer classification based on chromatin accessibility profiles with deep adversarial learning model
Source: PLoS Comput Biol. 2020 Nov 9;16(11):e1008405. doi: 10.1371/journal.pcbi.1008405 (PMC7676699; doi:10.1371/journal.pcbi.1008405)
Supplement: S3 Text — (DOCX) [file pcbi.1008405.s013.docx]

## S3 Text: The implementation details of GMM and random forest

We implemented the Gaussian Mixture module with the scikit-learn framework[1]. We set the number of clusters with K and set other parameters with the default values recommended by the toolbox. The initial mean and variance values of the mixed Gaussian distribution were derived from the K-means method, and the variation followed a diagonal-matrix mode. The convergence threshold was set to 1e-3.

We implemented the random forest with the scikit-learn framework [1]. The input of the model is the original ultra-high-dimensional data and the clustering labels of ClusterATAC, and the output is the Gini importance values of chromatin regions. The number of trees in the random forest is set to 100, and the maximum depth of the tree is set to 2.

Reference

1. Pedregosa F, Varoquaux G, Gramfort A, Michel V, Thirion B, Grisel O, et al. Scikit-learn: Machine Learning in Python. J Mach Learn Res. 2011;12:2825-30. PubMed PMID: WOS:000298103200003.
